# Promoting physical activity-related health competence to increase leisure-time physical activity and health-related quality of life in German private sector office workers

**DOI:** 10.1186/s12889-023-15391-7

**Published:** 2023-03-11

**Authors:** Simon Blaschke, Johannes Carl, Klaus Pelster, Filip Mess

**Affiliations:** 1 Department of Sport and Health Sciences, Associate Professorship of Didactics in Sport and Health, Technical University of Munich, Georg-Brauchle-Ring 60/62, Munich, 80992 Germany; 2 Department of Sport Science and Sport, Chair of Exercise and Health, Friedrich-Alexander University Erlangen-Nürnberg, Gebbertstraße 123b, Erlangen, 91058 Germany; 3 Siemens AG, Environmental Protection, Health Management and Safety – Health Management (P&O EHS DE HM), Lyoner Str. 27, Frankfurt am Main, 60528 Germany; 4 Department of Sport and Health Sciences, Associate Professorship of Didactics in Sport and Health, Technical University of Munich, Georg-Brauchle-Ring 60/62, 80992 Munich, Germany

**Keywords:** Health literacy, Physical literacy, Office workers, Health-related quality of life, Office workers, Behavioural change, Workplace health promotion

## Abstract

**Background:**

Office workers (OWs) are at risk of low levels of health-enhancing physical activity (HEPA) and impaired health-related quality of life (HRQOL). Interventions based on physical activity-related health competence (PAHCO) aim to facilitate long-term changes in HEPA and HRQOL. However, these assumptions rely on the changeability and temporal stability of PAHCO and have not been tested empirically. This study therefore aims to test the changeability and temporal stability of PAHCO in OWs within an interventional design and to examine the effect of PAHCO on leisure-time PA and HRQOL.

**Methods:**

Three hundred twenty-eight OWs (34% female, 50.4 ± 6.4 years) completed an in-person, three-week workplace health promotion program (WHPP) focusing on PAHCO and HEPA. The primary outcome of PAHCO as well as the secondary outcomes of leisure-time PA and HRQOL were examined at four measurement points over the course of 18 months in a pre-post design by employing linear mixed model regressions.

**Results:**

PAHCO displayed a substantial increase from the baseline to the time point after completion of the WHPP (β = 0.44, *p* < 0.001). Furthermore, there was no decrease in PAHCO at the first (*p* = 0.14) and the second follow-up measurement (*p* = 0.56) compared with the level at the end of the WHPP. In addition, the PAHCO subscale of PA-specific self-regulation (PASR) had a small to moderate, positive effect on leisure-time PA (β = 0.18, *p* < 0.001) and HRQOL (β = 0.26, *p* < 0.001). The subscale of control competence for physical training (CCPT) also had a positive small to moderate effect on HRQOL (β = 0.22, *p* < 0.001).

**Conclusion:**

The results substantiate PAHCO’s theoretical characteristics of changeability and temporal stability, and underline the theoretically postulated effects on leisure-time PA and HRQOL. These findings highlight the potential of PAHCO for intervention development, which can be assumed to foster long-term improvements in HEPA and HRQOL in OWs.

**Trial registration:**

The study was retrospectively registered in the German Clinical Trials Register, which is an approved Primary Register in the WHO network, at the 14/10/2022 (DRKS00030514).

**Supplementary Information:**

The online version contains supplementary material available at 10.1186/s12889-023-15391-7.

## Background

Globalisation and persistent advances in information as well as communication technologies have shaped the modern working world. Also, as a result of these advances, the number of office workers (OWs) has steadily increased in recent decades in western societies [1, 2] and represents, for example, a share of more than 40% of all employees in Germany [3]. OWs are primarily engaged in desk-based and digitally assisted on-site or remote occupational activities, such as reading, preparing and giving online or onsite presentations [4].

Partially as a result of these typically inactive and mostly digital occupational activities, OWs are exposed to an increased risk of developing chronic diseases, such as mental disorders [5], musculoskeletal disorders [6] or metabolic syndrome [7]. One crucial indicator for the prevention of chronic diseases [8, 9] is individuals’ health-related quality of life (HRQOL), which considers individuals’ perceived physical, mental, and social health status [10]. Alongside HRQOL as an indicator for detecting mental disorders and chronic diseases [11], HRQOL in OWs is positively linked to work-related measures, including higher employee productivity [12] and lower sickness absenteeism [13]. These findings highlight the importance of HRQOL as a health indicator for OWs and as an indicator for organisational outcomes.

To promote HRQOL, the World Health Organisation (WHO) addresses the health behaviour of individuals and specifically underlines the value of physical activity (PA) [14]. This public health strategy might be especially relevant for OWs, as Biernat and Piatkowska [15] report that fewer than 50% of OWs fulfil the WHO recommendation of at least 150 minutes of moderate-intensity or 75 minutes of vigorous-intensity PA per week [16]. Moreover, OWs show a lower amount of light and moderate PA and accumulate more sedentary time in comparison with other occupational groups [17]. In addition, OWs do not compensate for the high amount of work-related sedentary time by engaging in longer periods of leisure-time PA [18], which increases the risk of diminished HRQOL [19]. Next to reducing sedentary behavior and the overall need to increase the PA levels in OWs, precisely targeting health-enhancing PA (HEPA) might be crucial, as occupational PA – in contrast with leisure-time PA [15] – shows no or even an inverse relationship with HRQOL in OWs [20, 21]. HEPA comprises all types of PA that benefit health without causing undue harm or risk [22]. The potential of HEPA promotion in OWs is also indicated by a current review, which demonstrates a large positive effect of HEPA interventions on HRQOL in this target group [11]. However, owing to short follow-up periods in most of the studies included, as well as great heterogeneity across the applied interventions, Nguyen et al. [11] provide no detailed insights with regard to long-term benefits of HEPA interventions or HEPA intervention development in OWs. In summary, the relevance of OWs in the working population and the risk of impaired HRQOL in this occupational group along with the potential of HEPA highlight the need to promote HEPA among OWs.

Whereas these findings illustrate the potential of HEPA promotion in OWs, explaining PA behaviour is intricate [23, 24]. The complexity of explaining this behaviour is particularly evident for the maintenance of PA [25], with relapse posing a common challenge in this health behaviour [26]. Along with social and environmental factors, current studies substantiate the importance of individual factors, such as dispositions, attitudes and expectations, to change [27] and maintain PA [28]. The specific value of these individual factors towards increasing HEPA is also highlighted in the WHO’s “Global Action Plan on Physical Activity 2018–2030” [29], which underscores the importance of promoting competencies, physical literacy and health literacy, in order to increase individuals’ HEPA.

**Fig. 1:**
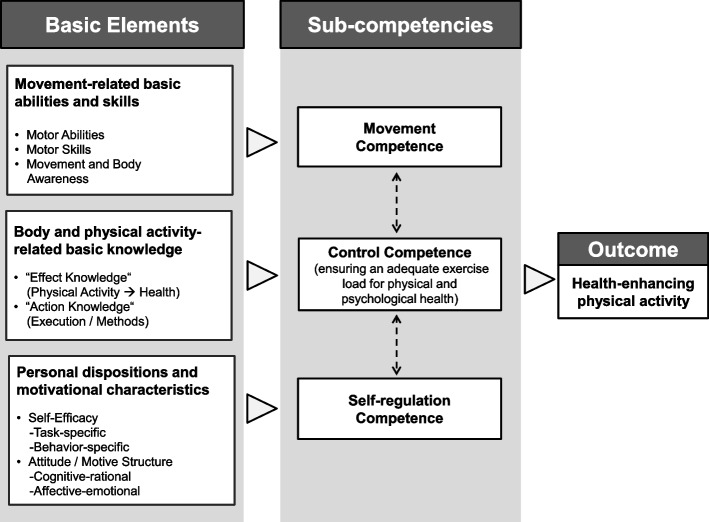
The Physical Activity-related Health Competence (PAHCO) model [32]

The Physical Activity-related Health Competence (PAHCO) model specifies competencies that are required in order to lead a physically active, healthy lifestyle and can be placed at the scientific intersection of physical literacy and health literacy [30, 31]. PAHCO consists of three integrated sub-competencies, which facilitate a physically active, healthy lifestyle (see Fig. 1) [32]. First, movement competence comprises direct motor-related requirements, which enable individuals to participate in planned exercise sessions (e.g. cycling and swimming) and to perform the activities of daily living (e.g. lifting heavy objects) [30]. Second, control competence ensures that PA achieves gains for health and is divided into a physical component consisting of the task to apply an adequate load and intensity (control competence for physical training – CCPT) and a psychological component for mental health benefits (PA-specific affect regulation; PAAR) [33]. Third, PA-specific self-regulation (PASR) includes psychological dispositions and motivational-volitional requirements to guarantee regular performance of physical activities [34].

In turn, these three sub-competencies arise from the specific coupling and integration of basic elements for PA behaviour [32]. Movement competence derives from motor abilities, motor skills as well as movement and body awareness (e.g., endurance, strength and balance). The sub-competence of control competence relies on individuals’ action knowledge and effect knowledge of PA to structure PA exercise subsequently in respect of the desired health-related outcome. PA-specific self-regulation is composed of self-efficacy, beneficial motive structures and positive attitudes toward PA.

In addition to the basic elements and sub-competencies that foster a physically active, healthy lifestyle, the authors of this model postulate that PAHCO holds the characteristics of a personality trait [30]. In line with modern personality trait theories [35] and competence research in educational sciences [36], PAHCO is assumed to display both changeability and relative temporal stability, which includes the potential for lasting promotion through structured interventions. Such interventions should address one or – following the idea of the integration of skills, knowledge and attitudes – ideally multiple basic elements to target the sub-competencies of the PAHCO model [30]. The possibility of promoting and maintaining PAHCO through interventions consequently holds the potential to facilitate long-term promotion of HEPA and HRQOL in OWs [37] and to mitigate the behavioural and work-related health risks of this occupational group.

Several empirical studies endorse the theoretical assumptions of this model with respect to their relationship to indicators of HEPA and HRQOL by displaying positive connections with PAHCO in various adult samples [31, 38, 39], with one study focusing on the target group of OWs [20]. On the sub-competence level, PA-specific self-regulation competence shows strong connections with leisure-time PA [20, 31, 39], whilst control competence for physical training demonstrates a moderate to strong connection with indicators of HRQOL in non-clinical adult samples [20, 38]. In line with the theoretical assumptions of the PAHCO model, these findings indicate the particular relevance of PA-specific self-regulation for regular participation in PA and point towards the value of control competence to facilitate effective HEPA and improvements in HRQOL.

However, the findings regarding the effect of PAHCO on HEPA and HRQOL remain tentative, as most of the research on this model has been cross-sectional [20, 31, 32, 34, 37, 38] with only few studies employing a longitudinal design in adult populations [39, 40]. Alongside this, two interventional studies [41, 42] yielded initial results concerning the changeability of this model in secondary school students showing improvements in PAHCO. Yet the assumption of changeability was unsupported in vocational students, who demonstrated no changes in PAHCO following a co-created PA intervention [43]. In addition to these mixed results with respect to changeability, Schmid et al. [39] investigated the temporal stability of PAHCO in a person-oriented approach over a period of 4 months and supported the authors’ assumptions of time-stable PAHCO sub-competencies [30]. In summary, Rosenstiel et al. [41] found no stable increase in PAHCO in secondary school students eight to 12 weeks after a physical education intervention but demonstrated a higher stability of PAHCO patterns in the students assigned to the intervention group in comparison with the control group students. This result might indicate higher temporal stability of PAHCO resulting amongst others from intervention components. However, the temporal stability of PAHCO could not be substantiated in an interventional study on PAHCO in secondary school students, which indicated initial changes in PAHCO after an intervention but no universally stable effects on PAHCO after 3 months [42]. While the findings of these studies are inconclusive in respect of PAHCO’s temporal stability over shorter periods, temporal stability over a longer time frame beyond 4 months has not yet been investigated. In addition to this research gap on temporal stability over longer periods, investigating PAHCO’s temporal stability over longer periods and the changeability of this construct resulting from HEPA interventions in OWs would empirically corroborate the conceptual assumptions of this model against the backdrop of modern personality trait theory and general competence research [35, 36]. The empirical examination of these assumptions is of particular relevance for the development of PAHCO interventions in OWs, as the changeability and temporal stability of PAHCO represents the cornerstone of this model for long-term improvements in HEPA and successful transfer for the promotion of HRQOL [33, 37, 39, 42].

The theoretical potential of PAHCO to target the need for promoting HEPA and HRQOL in OWs, in combination with the dearth of longitudinal research on this model, underscores the relevance to testing changeability and temporal stability of the sub-competencies as important conceptual assumptions of the PAHCO model. More specifically, the relevance with respect to temporal stability is particularly indicated for the period beyond 4 months, which was the maximum follow-up period investigated in previous studies. Owing to the importance of these theoretical assumptions for the development of interventions, the first research goal of this study concerns changeability and temporal stability of PAHCO and the sub-competencies of this model in OWs over the course of 18 months, following an in-person, three-week HEPA intervention. The second aim of the present study is to add empirical evidence regarding the postulated effect of the PAHCO subscales on HEPA and HRQOL over the period of 18 months. This second research goal will extends previous cross-sectional findings concerning the connections between PAHCO, HRQOL and indicators of HEPA over the course of the in-person intervention and the 18 month follow-up period.

## Methods

### Study design

This study was part of a project to evaluate the occupational health management system of a large, global, private-sector company in Germany from December 2020 to June 2022. The company’s employees received information about the aims of the study and the scope of the evaluation project orally, by email or from brochures. The participants of the WHPP were asked to complete the survey on paper at the start and after completion of the intervention, as well as online at follow-up measurements six and 18 months after the intervention. Identification of the participants across the four measurement times was achieved by means of an individual pseudonym, which was presented before completing the questionnaires.

The participants of this study were asked to provide their informed consent at the start of the intervention and before completing the online version of the survey at the follow-up stages. This study complied with the company’s data privacy guidelines. The Ethics Committee of the School of Medicine at the Technical University of Munich gave its ethical approval for this study (IRB number: 645/20 S-KH). The study was registered in the German Clinical Trials Register (DRKS00030514).

### Intervention description

The WHPP was developed by a German private-sector company to increase HEPA and HRQOL in employees and was held in-person at a wellness hotel in the district of Hof, Bavaria, Germany. The program of this WHPP was delivered over a period of 3 weeks by three exercise therapists, a physician and a psychologist, with a maximum of 60 participants per program. The intervention content of the WHPP is reported in accordance with the behaviour change technique taxonomy of Michie et al. [44].

At the start of the WHPP, the physician conducted a 60-minute initial physical examination and consultation to determine the health status and to advise on PA intensity as well as exercise session content in cooperation with the exercise therapist and the participant. Among other things, this medical screening served to inform participants about health consequences and to set personalised participative HEPA behavioural goals for the intervention period. The participants received a printed summary of the physical examination, which displayed e. g. blood pressure and triglycerides as well as the goals for HEPA during the intervention determined amongst others by physical working capacity test. On the next day, the exercise therapists introduced the participants to the indoor, outdoor and water aerobic exercise facilities in three 90-minute sessions and the participants took part in one 90-minute workshop focusing on the connection between PA and health, which was run by the physician. These sessions provided the participants with instructions and demonstrations of exercise behaviour and basic knowledge of HEPA.

Over the following 15 days, the participants followed a structured program, which comprised a 45-minute running or walking workout in the morning and a 90-minute exercise session after breakfast, both guided by the exercise therapists. The psychologist delivered a 90-minute relaxation technique session (e.g. autogenous training) or HEPA workshop after lunch. In the evening, the participants could voluntarily spend their leisure-time in the wellness clinic facilities or attend the psychologist’s presentations on the relationship between mental health and PA. The exercise therapists ran guided hiking trips for the participants near the wellness clinic twice a week in the afternoons over the course of the WHPP. There were no sessions on the three Sundays during the WHPP but the training facilities were accessible to all individuals. Participants focused on learning different types of HEPA (e.g. nordic walking, swimming, functional training) depending on their personal preferences and medical profile. Furthermore, they received information on the current state of knowledge with respect to biomechanics, behavioural psychology and exercise science. This phase of the WHPP was shaped by the participants’ behavioural practice with respect to HEPA, feedback on HEPA behaviour and instruction to self-monitor HEPA behaviour by the exercise therapists, as well as social support from other participants. This process was supported by the combination of theoretical and practical intervention components as well as by an interprofessional approach, which included HEPA and HRQOL from a psychological, medical and exercise science perspective [45].

In the last week, the physician conducted a second physical examination to screen changes in the participants’ medical profile (e.g. blood pressure and trigylcerides) and to set participative goals for maintaining HEPA after completion of the WHPP. The physicians and exercise therapists gave instructions for the participants’ goal setting and action planning as well as problem solving strategies. Furthermore, the participants refined HEPA behaviour implementation intentions in workshops with the exercise therapist and psychologist on the last 2 days of the WHPP. This phase of the WHPP focused on transferring HEPA behaviour change into the participants’ daily lives, so that HEPA behaviour could be maintained. The entire curriculum, material overview and WHPP schedule can be accessed by contacting the corresponding author.

### Sampling procedure and description

Before participants were recruited, the minimum sample size was calculated using a repeated measures within-between interaction analysis of variance design in GPower 3.1 [46]. According to this calculation, a sample size of *N* = 98 was needed to detect an effect of *f*^*2*^ = 0.20 with the statistical power of 1-β = 0.95 and a type one error of *p* = 0.05 under the assumption of repeated measures correlation of *r* = 0.50 at four measurement times. This calculation served as the lower threshold for data collection with attrition rates in longitudinal studies ranging from 30 to 70% [47].

**Fig. 2:**
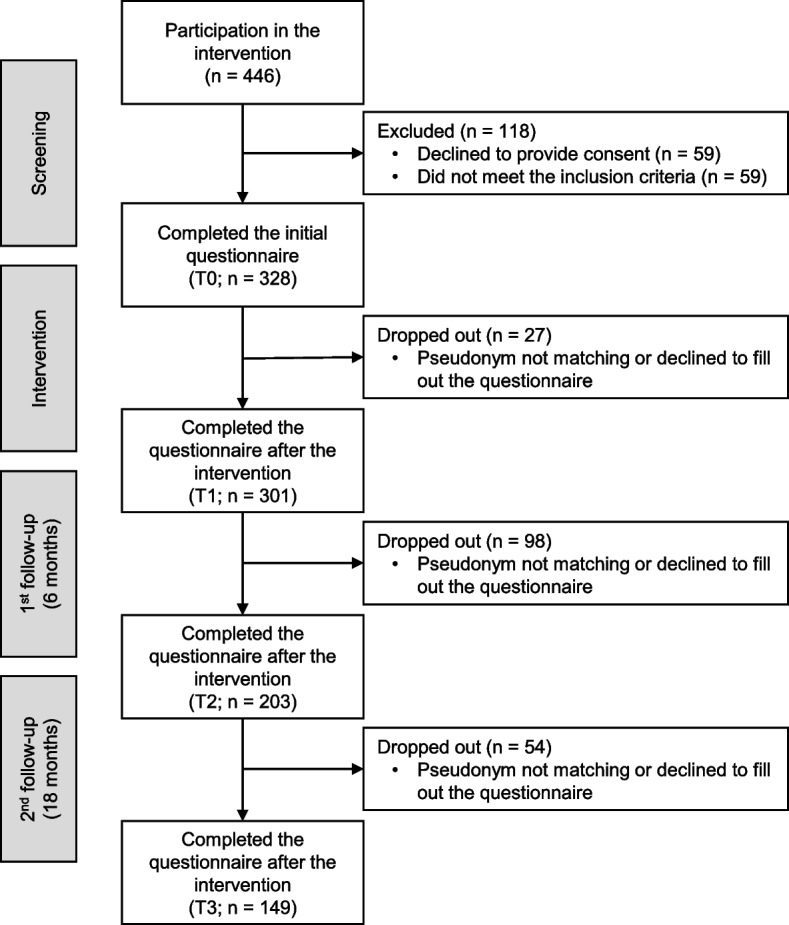
Overview of participant enrolment and drop out over the course of the data collection period

During the data collection period, 446 employees took part in the intervention. Three hundred eighty-seven employees registered their interest to participate in the study. 328 (85%) participants met the first inclusion criterion of being engaged in office work occupation, and 45 (12%) people were ineligible owing to assembly work tasks; a further 14 (4%) were ineligible owing to construction work duties. All 328 participants met the second inclusion criterion, i.e. absence from acute mental or physical disease, and completed the survey at the start of the intervention. In total, 149 (39%) participants completed the survey after the intervention as well as at the first follow-up measurement after 6 months and the second follow-up measurement after 18 months. A detailed overview of participant enrolment is shown in Fig. 2.

### Measures

The participants gave information on their sociodemographic status by reporting their gender, age, relationship status and educational level. To assess the primary and secondary outcomes, this study utilised valid and reliable instruments for the population of German adults [32, 48, 49].

The primary outcome of this study was assessed with the PAHCO questionnaire developed by Sudeck and Pfeifer [31]; consisting of the three subscales control competence for physical training (CCPT), PA-specific affect regulation (PAAR) and PA-specific self-regulation (PASR), with a total of 13 items rated on a 4-point Likert scale. The first subscale, CCPT (e.g. “If my muscles are tensed up, I know exactly how to counter this through physical activity”), contains six items (Cronbach’s α = 0.86). The second subscale, PAAR (e.g. “I am able to regulate my mood through physical activity”), comprises four items (Cronbach’s α = 0.82). The third subscale, PASR (e.g. “I stick with my plan to do exercise and am not easily distracted from that plan”), comprises three items (Cronbach’s α = 0.84). Mean scores are calculated for the three subscales and for all 13 items of the PAHCO questionnaire, with values ranging from one to four and higher mean scores indicating better PAHCO (Cronbach’s α = 0.92).

The secondary outcome, HEPA, was operationalised with the Godin-Shepard Leisure-Time Physical Activity Questionnaire (GSLTPAQ) [50]. This questionnaire examines PA during leisure time at light, moderate and vigorous intensities (e. g. “Over the last 7 days (i.e., the last week), how many times on average did you do the following kinds of exercise for more than 30 min during your free time?”) resulting in the cumulative weighted leisure score index (LSI). In agreement with the procedure of calculating LSI in healthy adults proposed by Amireault et al. [51], LSI was examined by multiplying the bouts at moderate intensity by five and nine at vigorous intensity, and adding up the two products of this calculation. The values of the LSI start at zero, and values above 24 indicate sufficient leisure-time PA [51].

HRQOL was examined using Short-Form Health Survey (SF-12) version 2.0 [10, 49]. SF-12 version 2.0 has a total of twelve items, which examine HRQOL with a weighted and standardised component score in a physical (Cronbach’s α = 0.80) and a mental (Cronbach’s α = 0.86) dimension. For the total score on HRQOL, the mean of the two component scores was calculated. The scores on HRQOL range from zero to 100, with a mean (*M*) of 50 and a standard deviation (*SD*) of 10; high values indicate better HRQOL.

### Statistical analysis

The research questions of this study were tested in a per-protocol (PP) analysis, which included all participants that remained within the main study until completion of the follow-up [52]. PP analysis serves to investigate the potential efficacy of the intervention for participants who adhere to the study protocol [53], as the research questions addressed the theoretical assumptions of PAHCO and not the effectiveness of the intervention, which is typically analysed by an intention-to-treat approach. Data preparation as well as descriptive and inferential statistical analyses were performed with R and RStudio (Version 4.2.1; RStudio Inc., Boston, MA, USA) [54].

In the data preparation process the handling of low quality data, for example due to illogical LSI scores, was operationally defined by the first author, approved by the authors of this study and excluded from the analysis. If all authors were unsure about the exclusion of potentially unreasonable survey responses the data was retained. Missing data, for example owing to a partially incomplete survey response, were imputed by applying multivariate-chained equations, when the assumption of data missing at random was met [55]. Multivariate outliers were excluded before the analysis, using Mahalanobis distance, based on the recommendations by Tabachnick and Fidell [56]. The assumptions of linearity, normality, homoscedasticity and independence of the residuals were examined on the basis of current guidelines for linear regression analyses [57].

Four linear, mixed-model (LMM) regressions, one for the total PAHCO score and three for the PAHCO subscales, were run with the participants’ pseudonym as the random intercept, to test the first research goal of changeability and temporal stability of PAHCO [58]. For the second research goal, the secondary outcomes of leisure-time PA and HRQOL were tested separately over the four measurement times, with the three PAHCO subscales as fixed effects and the participants’ pseudonym as a random effect. The measures of HRQOL, leisure-time PA, age, gender, relationship status and educational level served as fixed effects in the LMM regressions.

Standardised estimates (β), confidence intervals (*CI 95%*) and *p*-values were examined to determine the influence of the fixed effects on the outcome. The final regression models are presented in comparison with the null model with Akaike’s Information Criteria (*AIC*), Bayesian Information Criteria (*BIC*) and adjusted R-squared (*R*^*2*^), on the basis of one imputed data set. Tukey’s post hoc test was used to test the temporal stability of PAHCO and the models’ subscales at the first and second follow-up measurement compared with the level after HEPA intervention completion [59]. Standardised estimates of the LMM analyses and post hoc tests were interpreted as small (≈0.10), moderate (≈0.30) or strong effects (≈0.50) [60]. On the basis of multiple testing, we adjusted the significance level of the LMM analyses and post hoc testing to *p* < 0.01 [61].

## Results

### Basic descriptive analyses

The assumptions of linearity, normality, homoscedasticity and independence of the residuals were maintained before the regression analyses. The sociodemographic characteristics of the sample at baseline are shown in Table 1. From the 328 participants at baseline, 149 (39%) participants were included in the PP analysis to answer by completing the survey after the intervention as well as at the first follow-up measurement and the second follow-up measurement.

**Table 1:** Sociodemographic characteristics of the participants

	Total (***N*** = 328)	Men (***n*** = 216)	Women (***n*** = 112)
Age, M (SD)	50.4 (6.4)	50.7 (6.5)	49.7 (6.1)
Relationship status			
Relationship, n (%)	240 (73.2)	170 (78.7)	70 (60.5)
No relationship, n (%)	88 (26.8)	46 (21.3)	42 (37.5)
Education			
Tertiary, n (%)	200 (61.0)	155 (71.8)	45 (40.2)
Secondary, n (%)	114 (34.7)	56 (25.9)	58 (51.8)
Primary, *n* (%)	14 (4.3)	5 (2.3)	9 (8.0)

*M* Mean, *SD* Standard deviation, *N* Total sample, *n* Subsample

Descriptive statistics (*M* and *SD*) for the overall PAHCO score and the PAHCO subscales, the HEPA indicator and the HRQOL score across the four measurement times are shown in Table 2. The descriptive analysis points towards an improvement in the primary and secondary outcomes from the first to the second measurement point. Descriptively, the primary outcomes of PAHCO and the respective subscales remain relatively constant at the first and the second follow-up measurement in comparison with the level after completion of the intervention. While PAAR seems to be descriptively most stable temporarily after the intervention, PASR displays the biggest decrease in the descriptive analysis. In contrast, the secondary outcomes of leisure-time PA and HRQOL show a more substantial descriptive decline at the follow-up measurement time points compared with the level after completion of the intervention. In particular, leisure-time PA descriptively shows a substantial decrease after the intervention.

**Table 2:** *M* and *SD* of the primary and secondary outcome parameters for the measurement points

		T0	T1	T2	T3
**PAHCO**	*M*	2.68	3.26	3.22	3.17
	*SD*	0.43	0.39	0.43	0.46
**CCPT**	*M*	2.62	3.27	3.27	3.22
	*SD*	0.56	0.40	0.45	0.48
**PAAR**	*M*	2.80	3.14	3.15	3.17
	*SD*	0.61	0.59	0.58	0.58
**PASR**	*M*	2.62	3.34	3.24	3.11
	*SD*	0.62	0.57	0.63	0.71
**Leisure-time PA**	*M*	23.49	84.23	36.52	32.90
	*SD*	17.05	34.48	20.99	19.57
**HRQOL**	*M*	47.24	54.88	51.14	50.72
	*SD*	6.09	3.75	5.47	5.48

*M* Mean, *SD* Standard deviation, *T0* Start of the program, *T1* End of the program, *T2* 6-month follow-up, *T3* 18-months follow-up, *PAHCO* Physical Activity-Related Health Competence, *CCPT* Control Competence for Physical Training, *PAAR* Physical Activity-Specific Affect Regulation, *PASR* Physical Activity-Specific Self-Regulation, *HRQOL* Health-Related Quality of Life

### Analysing the changeability and temporal stability of PAHCO

A detailed overview of the results of the analysis with respect to PAHCO’s changeability can be found in Table 3. Temporal stability of PAHCO is shown in Table 4. The description of the LMM regressions and the model fit parameters are presented in Supplementary Table 1.

**Table 3:** Results of the LMM regressions for PAHCO score and PAHCO subscales over time

	Criterion					
	**PAHCO**			**CCPT**		
**Predictor**	***SE***	**β**	***CI 95%***	***SE***	**β**	***CI 95%***
**Gender**						
**Male**	0.07	0.04	−0.09 / 0.18	0.07	−0.00	− 0.14 / 0.13
**Age**	0.03	0.02	−0.03 / 0.08	0.03	0.03	−0.04 / 0.09
**Educational Level**						
**Secondary**	0.17	0.29	−0.03 / 0.62	0.20	0.20	−0.19 / 0.60
**Tertiary**	0.17	0.29	−0.04 / 0.61	0.20	0.30	−0.10 / 0.70
**Relationship Status**						
**Current Relationship**	0.06	−0.05	−0.17 / 0.06	0.07	−0.08	− 0.20 / 0.05
**Leisure-Time PA**	**0.02**	**0.25**	**0.15 / 0.36**	0.03	0.05	−0.01 / 0.10
**Time**						
**T1**	**0.05**	**0.44**	**0.33 / 0.54**	**0.06**	**0.56**	**0.44 / 0.68**
**T2**	**0.04**	**0.51**	**0.44 / 0.57**	**0.04**	**0.63**	**0.54 / 0.71**
**T3**	**0.04**	**0.46**	**0.39 / 0.53**	**0.04**	**0.59**	**0.50 / 0.67**
	**PAAR**			**PASR**		
**Predictor**	***SE***	**β**	***CI 95%***	***SE***	**β**	***CI 95%***
**Gender**						
**Male**	0.11	0.10	−0.12 / 0.32	0.10	0.06	−0.14 / 0.25
**Age**	0.04	−0.01	− 0.08 / 0.08	0.04	0.02	−0.07 / 0.10
**Educational Level**						
**Secondary**	0.28	0.53	−0.03 / 0.91	0.25	0.23	−0.25 / 0.72
**Tertiary**	0.28	0.49	−0.06 /0.87	0.25	0.16	−0.32 / 0.65
**Relationship Status**						
**Current Relationship**	0.09	−0.02	−0.20 / 0.17	0.09	−0.05	− 0.23 / 0.13
**Leisure-Time PA**	0.06	0.06	− 0.07 / 0.20	**0.04**	**0.16**	**0.09 / 0.23**
**Measurement Time**						
**T1**	**0.07**	**0.33**	**0.19 / 0.47**	**0.08**	**0.40**	**0.24 / 0.57**
**T2**	**0.05**	**0.36**	**0.27 / 0.45**	**0.06**	**0.53**	**0.42 / 0.63**
**T3**	**0.05**	**0.38**	**0.29 / 0.47**	**0.06**	**0.44**	**0.33 / 0.54**

*PAHCO* Physical Activity-Related Health Competence, *CCPT* Control Competence for Physical Training, *PAAR* Physical Activity-Specific Affect Regulation, *PASR* Physical Activity-Specific Self-Regulation, *T0* Start of the intervention, *T1* End of the intervention, *T2* 6-month follow-up, *T3* 18-month follow-up, Reference group for Gender is ‘Female’, Reference group for Educational Level is ‘Primary’, Reference group for Relationship Status is ‘No relationship’, Reference group for Measurement Time is ‘T0’, Results with a *p-*value < .01 are shown in bold, *SE* Standard error, *β* Standardised regression estimate, *CI 95%* 95% confidence interval

**Table 4:** Temporal stability of PAHCO and the subscales after completion of the intervention

	Criterion					
	**PAHCO**			**CCPT**		
**Difference**	***SE***	**β**	***CI 95%***	***SE***	**β**	***CI 95%***
**T2 – T1**	0.05	−0.07	− 0.16 / 0.02	0.06	− 0.07	− 0.18 / 0.05
**T3 – T1**	0.05	−0.03	− 0.12 / 0.07	0.06	− 0.03	− 0.14 / 0.09
**T3 – T2**	0.03	0.04	−0.03 / 0.11	0.04	0.04	−0.04 / -0.12
	**PAAR**			**PASR**		
**Predictor**	***SE***	**β**	***CI 95%***	***SE***	**β**	***CI 95%***
**T2 – T1**	0.06	−0.03	− 0.15 / 0.09	0.07	− 0.13	− 0.26 / 0.03
**T3 – T1**	0.07	−0.05	− 0.18 / 0.08	0.08	−0.04	− 0.17 / 0.13
**T3 – T2**	0.05	−0.02	− 0.11 / 0.07	0.05	0.09	−0.01 / 0.20

*PAHCO* Physical Activity-Related Health Competence, *CCPT* Control Competence for Physical Training, *PAAR* Physical Activity-Specific Affect Regulation, *PASR* Physical Activity-Specific Self-Regulation, *T1* End of the intervention, *T2* 6-month follow-up, *T3* 18-month follow-up, Results with a *p-*value < .01 are shown in bold, *SE* Standard error, β Standardised regression estimate, *CI 95%* 95% confidence interval

The LMM regression to test the first research goal, which investigated the changeability and temporal stability of PAHCO and the subscales of this model, showed a substantial increase for the total PAHCO score after completion of the HEPA intervention (β = 0.44, *CI 95%* [0.33, 0.54], *p* < 0.001) as well as after the first (β = 0.51, *CI 95%* [0.44, 0.57], *p* < 0.001) and second follow-up measurement (β = 0.46, *CI 95%* [0.39, 0.53], *p* < 0.001). In the post hoc analyses, PAHCO revealed neither a drop-off at the first follow-up measurement (*p* = .18) nor a decrease at the second follow-up measurement (*p* = .61) in comparison with the level after completion of the HEPA intervention.

On the subscale level, control competence for physical training (CCPT) displayed substantial gains in comparison with the baseline level after the HEPA intervention (β = 0.56, *CI 95%* [0.44, 0.68], *p* < 0.001) at the first follow-up (β = 0.63, *CI 95%* [0.54, 0.71], *p* < 0.001) and second follow-up measurement (β = 0.59, *CI 95%* [0.50, 0.67], *p* < 0.001). CCPT showed no decrease in the post hoc analyses after completion of the HEPA intervention at the first follow-up measurement (*p* = 0.26) and second follow-up measurement (*p* = 0.70).

The LMM regression of the subscale of PA-specific affect regulation (PAAR) showed a moderate, positive increase after completion of the HEPA intervention (β = 0.33, *CI 95%* [0.19, 0.47], *p* < 0.001), at the first follow-up measurement (β = 0.36, *CI 95%* [0.27, 0.45], *p* < 0.001) and the second follow-up measurement (β = 0.38, *CI 95%* [0.29, 0.47], *p* < 0.001) compared with the baseline. The post hoc analyses revealed no decrease in PAAR at the first (*p* = 0.64) and second follow-up measurement (*p* = 0.46) compared with the PAAR level after completion of the HEPA intervention.

The subscale of PA-specific self-regulation (PASR) displayed a positive, moderate increase after the HEPA intervention (β = 0.40, *CI 95%* [0.24, 0.57], *p* < 0.001). This subscale showed a strong gain at the first follow-up measurement (β = 0.53, *CI 95%* [0.42, 0.63], *p* < 0.001) and a moderate gain at the second follow-up measurement (β = 0.44, *CI 95%* [0.33, 0.54], *p* < 0.001) in comparison with the baseline scores in the LMM. The post hoc analyses showed no decrease in this subscale at the first follow-up (*p* = 0.09) and second follow-up measurement (*p* = .65) in comparison with the level after completion of the HEPA intervention.

### Analysis of the longitudinal effect of the PAHCO subscales on leisure-time PA and HRQOL

The analysis of the second research goal, which investigated an effect of the PAHCO subscales on leisure-time PA and HRQOL, is summarised in Table 5. PASR displayed a small to moderate, positive influence of leisure-time PA (β = 0.18, *CI 95%* [0.11, 0.25], *p* < 0.001). The subscales of CCPT (*p* = 0.85) and PAAR (*p* = 0.07) displayed no noteworthy effect on leisure-time PA. In addition, leisure-time PA did not display a significant effect 18 months after completing the intervention (*p* = 0.16).

**Table 5:** Results of the LMM regressions for leisure-time PA and HRQOL over time

	Criterion					
	Leisure-time PA			HRQOL		
Predictor	***SE***	β	***CI 95%***	***SE***	β	***CI 95%***
**Gender**						
**Male**	0.09	0.00	−0.07 / 0.07	0.12	−0.02	− 0.13 / 0.10
**Age**	0.04	−0.03	− 0.11 / 0.03	0.05	−0.11	− 0.23 / 0.00
**Educational Level**						
**Secondary**	0.25	0.39	−0.12 / 0.28	0.30	−0.52	−0.82 / -0.01
**Tertiary**	0.26	0.14	−0.07 / 0.34	0.29	−0.43	− 0.74 / 0.00
**Relationship Status**						
**Current Relationship**	0.09	0.00	−0.07 / 0.07	**0.12**	**0.22**	**0.12 / 0.33**
**Leisure-Time PA**				0.04	0.04	−0.06 / 0.15
**HRQOL**	0.04	0.03	−0.04 / 0.10			
**CCPT**	0.04	−0.01	− 0.09 / 0.07	**0.04**	**0.22**	**0.12 / 0.33**
**PAAR**	0.03	−0.02	−0.09 / 0.05	0.04	0.06	−0.04 / 0.16
**PASR**	**0.04**	**0.18**	**0.11 / 0.25**	**0.04**	**0.26**	**0.16 / 0.36**
**Time**						
**T1**	**0.09**	**0.72**	**0.65 / 0.80**	**0.12**	**0.40**	**0.28 / 0.52**
**T2**	0.08	0.17	0.03 / 0.17	0.09	0.11	0.02 / 0.20
**T3**	0.08	0.06	0.00 / 0.13	0.08	0.11	0.02 / 0.20

*HRQOL* Health-Related Quality of Life, *CCPT* Control Competence for Physical Training, *PAAR* Physical Activity-Specific Affect Regulation, *PASR* Physical Activity-Specific Self-Regulation, *T0* Start of the intervention, *T1* End of the intervention, *T2* 6-month follow-up, *T3* 18-month follow-up, Reference group for Gender is ‘Female’, Reference group for Educational Level is ‘Primary’, Reference group for Relationship Status is ‘No relationship’, Reference group for the Measurement Time is ‘T0’, Results with a *p-*value < .01 are shown in bold, *SE* Standard error, β Standardised regression estimate, *CI 95%* 95% confidence interval

With respect to the effect of PAHCO on HRQOL, the subscales of CCPT (β = 0.22, *CI 95%* [0.12, 0.33], *p* < 0.001) and PASR (β = 0.26, *CI 95%* [0.16, 0.36], *p* < 0.001) had a small to moderate, positive effect on the HRQOL. PAAR showed no notable connection with HRQOL (*p* = 0.23). HRQOL showed no significant effect following the intervention after six (*p* = 0.02) and 18 months (*p* = 0.01).). HRQOL showed no significant effect following the intervention after six (*p* = 0.02) and 18 months (*p* = 0.01).

## Discussion

### Explaining the changeability and temporal stability of PAHCO

The results of the analysis confirm the first research goal, which assumed changeability and temporal stability of PAHCO, by displaying a moderate to strong increase over the course of the WHPP as well as no decrease over the follow-up period of a total of 18 months.

Our findings are in line with results on the changeability of PAHCO in a sample of secondary school students, which demonstrated a moderate effect for the increase of PAHCO following a physical education intervention [42]. Additionally, our results also align with reviews on health literacy [62] and physical literacy [63] interventions, which substantiate the changeability of these constructs in working aged adults. Yet Grüne et al. [43] found no effects on PAHCO following a PA intervention in vocational students. The authors of this study in the school setting, however, discussed their results against the backdrop of implementation obstacles and potential theoretical inconsistencies in intervention development. In contrast with these limitations, our study might have improved PAHCO as a result of the high amount of guided HEPA behaviour and the face-to-face design of the intervention, highlighted as beneficial intervention components by a review on the promotion of PA motivation [64], which is integrated in the basic elements of PAHCO.

The findings of our study on temporal stability of PAHCO agree with theoretical research on PAHCO [30] by displaying no changes of PAHCO level 18 months after the intervention. These results are partially in line with results from Rosenstiel et al. [41], who underlined a higher temporal stability of PAHCO after intervention in physical education classes with secondary school students in comparison with the control group. However, this study did not indicate universally stable improvement following a physical education intervention in PAHCO after 8–12 weeks, which partially contradicts our results. While this study by Rosenstiel [41] also supposed temporal stability in PAHCO after the physical education intervention, the authors connected the lacking sustainability of the intervention effects with a small total number of six intervention sessions lasting 90 minutes each. The total number of intervention sessions in our study differed largely from this intervention design, which might explain different findings with respect to the stability of intervention effects in PAHCO at the follow-up measurements. This claim is supported by a review on PA [65], which underlines the importance of aspects connected with the duration of PA interventions, such as total contact time with the exercise therapists. In addition, the differing results on PAHCO’s temporal stability in the study on secondary school students by Rosenstiel [41], in comparison with our findings in OWs, could be due to lower temporal stability of PAHCO in younger populations. This idea is underlined by a review on trait stability, which postulates a consolidation of personality traits in young adulthood [66].

With respect to the longer follow-up period in comparison with previous PAHCO interventions, our findings are supported by a meta-analysis of interventions addressing PA motivation, which showed stable intervention effects at follow-up time points beyond 6 months [64]. In addition, current reviews suggest an overall positive effect of interventions on PA self-efficacy [67], health literacy [62] and physical literacy [63], which share much common ground with the PAHCO model. Yet the temporal stability of these interventions is small to negligible for PA self-efficacy [67] and uncertain for physical and health literacy, as most studies – including on these constructs – lack follow-up periods beyond 6 months or examine inconsistent results at these later measurement points [68, 69].

In summary, the findings of our study on PAHCO’s changeability and temporal stability substantiate the theoretical assumptions of this model, largely in line with the existing literature and extend the current knowledge on PAHCO. These results might be of particular importance for future WHPPs, as interventions on PAHCO could lead to temporarily stable changes in PAHCO, which consequentially could promote HEPA and HRQOL in OWs. The potential of temporarily stable changes in PAHCO might present a specific benefit for OWs, as this target group is prone to insufficient levels of PA at low and moderate intensity and to longer periods of sedentary, work-related activities [18]. In addition, these changes in PAHCO could also promote leisure-time PA, which would allow OWs to compensate for and change these work-related activity patterns. However, as this is the first study in non-clinical adults to examine the changes of PAHCO following an intervention, future research is needed to corroborate our findings.

### Explaining the longitudinal effect of the PAHCO subscales on leisure-time PA and HRQOL

The second research goal, which postulated a positive effect of the PAHCO subscales on leisure-time PA and HRQOL in OWs can be confirmed for the PASR subscale, which displayed a positive, small to moderate effect on leisure-time PA and HRQOL. Alongside this, the subscale of CCPT showed a positive small to moderate effect on HRQOL, which also supports the second research goal. Yet the subscales of CCPT and PAAR did not indicate an effect on leisure-time PA and PAAR showed no noteworthy longitudinal effect on HRQOL.

In general, although the measures on PA and HRQOL were deviating from our study, these findings are in agreement with cross-sectional studies on PAHCO, PA and HRQOL in adult populations [31, 39] as well as the target group of OWs [20]. These studies underline the importance of the subscales of CCPT and PASR in connection with leisure-time PA and HRQOL. To the best of our knowledge, only one study has previously investigated longitudinal relationships of PAHCO with PA and HRQOL [39] in a non-clinical adult population. Although our results are generally in line with its findings, this study had a person-oriented approach and investigated PAHCO over a period of 4 months, which impedes the integration of our findings into the current literature.

Alongside this person-oriented study, our findings are partly in agreement with the results of a longitudinal study on PAHCO in chronic obstructive pulmonary patients, which found a positive, bivariate relationship between the subscales of PAAR and PASR after a rehabilitation program and the PA level 6 months after the program [40]. In addition, this study also found a positive, bivariate relationship between the subscales of PASR, PAAR and CCPT after the rehabilitation program and the patients’ quality of life after 6 months. However, structural equation model analysis of PAHCO in this sample of chronic obstructive pulmonary patients revealed a connection between patients’ movement competence after the program and the outcomes of PA and quality of life after 6 months. The subscales of CCPT, PAAR and PASR showed no connection with PA and quality of life after 6 months in the structural equation model [40]. Although movement competence was not examined in our study, the differing results compared with the research by Carl et al. [40] might also derive from varying populations and study designs. Movement competence might, for example, display largely differing connections with PA in patients with chronic obstructive pulmonary diseases compared with OWs, because of the burden of disease-limiting possibilities for PA participation [70].

In addition, our findings on PAAR and HRQOL differ from the results found by Sudeck et al. [34], which focused on PAAR and proved a moderating effect of PAAR on the relationship of PA and mental aspects of HRQOL over a period of 4 days. PAAR, which covers individuals’ ability to achieve mental health benefits from PA, showed no effect on leisure-time PA and HRQOL in our study. These contradictions could be due to various differences between our study and the research by Sudeck et al. [34]. While the later research comprised a period of 4 days with a high proportion of physically active adults and employed different measures of PA and HRQOL, our analysis focused on a period of more than 18 months and a sample of mostly inactive OWs and did not include PAAR as a moderator for the PA-HRQOL connection. Future studies, however, might focus on the relationships of this subscale with PA and HRQOL in a longitudinal moderation analysis in OWs.

In addition to these studies, which address PAHCO directly, the results on the subscale of PASR are in line with reviews on PA-self efficacy [71] and PA motivation [64] in healthy adults, which demonstrate that interventions targeting these concepts are associated with higher levels of PA. In addition, the review by Medrano-Ureña et al. [72] found that PA self-efficacy interventions can also produce higher levels of HRQOL. The subscale of PASR shows substantial overlap with PA-self efficacy and PA motivation and concerns the theoretical assumption of increasing regular HEPA participation, which might in return promote HRQOL [30]. The effect of CCPT on HRQOL is also indicated by the review from Nguyen et al. [11], which focuses on exercise interventions, also addressing PA-related knowledge in OWs to promote HRQOL in OWs. Within this review, a large proportion of the interventions addressed participants’ knowledge on physical load or execution of HEPA before or during the intervention programs. These PA-related knowledge aspects are a central component of CCPT, which might explain the positive effect of this subscale on HRQOL in our study, also investigating OWs.

With regard to the connections of the closely related constructs of health and physical literacy with leisure-time PA and HRQOL, a review on health literacy and PA found positive connections between these concepts but no intervention effects of health literacy on PA [73]. Furthermore, health literacy interventions displayed a weak positive effect on health outcomes, such as HRQOL, but were also limited by insufficient study designs and heterogeneous health literacy interventions [62]. A current review by Carl et al. [63] supports the effectiveness of physical literacy on PA but found no studies focusing on the effect of physical literacy interventions on health. The shortcomings of health literacy interventions to promote PA as well as the lack of current physical literacy studies addressing health outcomes along with PA might substantiate the value of PAHCO, which theoretically combines benefits of both constructs.

### Strengths and limitations

This is the first study to explore PAHCO, leisure-time PA and HRQOL in OWs in a longitudinal design. The focus on OWs might be particularly important, owing to the high risk of physical inactivity in this population, which is largely due to the work-related demands of this occupation [17]. In addition, this study tries to overcome the shortcoming of a large proportion of longitudinal studies in PA promotion [74] and previous longitudinal studies on PAHCO [39] by employing a follow-up period beyond 6 months to examine PAHCO temporal stability, leisure-time PA maintenance and long-term HRQOL promotion 18 months after completion of the WHPP. Moreover, our study extends the theoretical and empirical knowledge of PAHCO by exploring the changeability and temporal stability of PAHCO in a non-clinical adult population, as well as analysing the effect of this model on leisure-time PA and HRQOL. These extensions to previous studies into the characteristics and effect of PAHCO are crucial to illustrate the potential of interventions that specifically target PAHCO in these populations [37].

Despite these strengths, this study has some limitations. The sub-competence of movement competence was not assessed within the version of the PAHCO questionnaire used in our study, as movement competence was not part of this measure at the time of data collection [32]. The absence of movement competence from the PAHCO questionnaire is derived from the complexity of valid operationalisation methods across various populations [75]. This problem was resolved by Carl et al. [32] in a study incorporating movement competence and validating the refined PAHCO questionnaire across various adult samples. Movement competence, which covers locomotor abilities and skills for PA in exercise and daily living situations, might explain additional variance of HEPA and HRQOL in our study with OWs, as indicated in longitudinal findings by Carl et al. [40] on PAHCO, PA and quality of life in chronic obstructive pulmonary disease patients.

In addition, the LSI might not be a sufficient tool to comprehensively examine HEPA as light and very light, non-leisure PA and active commuting are not included in the LSI scoring. These PA forms display a positive connection with health in current meta-analyses [76, 77] and therefore might have been included to precisely explore the HEPA-health relationship. In this study, these forms of PA were not implemented as the main focus was on the promotion of PAHCO and its effect on facets of leisure-time PA, which served as a proxy parameter for HEPA, and health. With previous studies, also in the target group of OW, displaying no relationship between occupational PA and health [20, 21] and another study showing no direct connection between overall PA and health [38], this study only employed the GSLTPAQ to examine a substantial facet of HEPA.

Furthermore, future research on PAHCO, PA, and HRQOL in OWs might benefit from objective measures of PA and health. In this regard, the present study might have been affected by subjective bias in the primary outcomes [78, 79]. For example, the use of accelerometers in the examination of PA would allow a more precise measure of this health behaviour, whilst also opening up the possibility of incorporating the assessment of sedentariness [80]. The advantage, which could result from objective measures of PA, might be crucial, because these health behaviours could also play a vital role in the relationship of PAHCO and health in OWs [81].

Finally, our study employed no control group or randomisation for the HEPA intervention. Therefore, the findings of this study do not offer causal conclusions on the effect of the intervention on PAHCO, leisure-time PA and HRQOL. Even though research in occupational settings is affected by a multitude of organisational and logistical problems, at times impeding the implementation of a controlled design [82], randomised control trials are needed to quantify the effect of HEPA interventions in the workplace on PAHCO without the potential of observation bias [83].

## Conclusion

This study extends current research into PAHCO by investigating the changeability and temporal stability of PAHCO as well as examining the effects of PAHCO on leisure-time PA and HRQOL in a sample of German OWs. While this population might be particularly prone to impaired HRQOL, owing to work-related physical inactivity, our findings underline the changeability and temporal stability of PAHCO, which implies important characteristics for intervention development to address the health-related demands and resources of this occupational group. The potential of PAHCO for the development of interventions in OWs is additionally substantiated, as our results suggest that motivational and volitional aspects of PAHCO can promote leisure-time PA and HRQOL, while aspects focusing on knowledge regarding the adaption of physical load during PA improve HRQOL. Based on these insights, comprehensive WHPPs could incorporate PAHCO by implementing components of HEPA-related knowledge in existing programs to facilitate health promotion and additionally refine the communication strategies to contain timely motivational and volitional cues, which could facilitate behaviour change and maintenance in OWs. In summary, these findings and the potential implications should, on the one hand, encourage practitioners to incorporate PAHCO within WHPPs to increase HEPA in OWs. On the other hand, future, randomised control trials on PAHCO in OWs should expand the field of study by including objective measures on PA, which would also account for sedentariness and test our results in respect of the potential of PAHCO in WHPPs.

## Supplementary Information

**Additional file 1: Supplementary Table 1.** Model-fit parameters for null model and the final regression models. **Supplementary Fig. 1.** Physical activity-related health competence over time. **Supplementary Fig. 2.** Control competence for physical training over time. **Supplementary Fig. 3.** Physical acitivity-specific affect regulation over time. **Supplementary Fig. 4.** Physical acitivity-specific self-regulation over time.

## Data Availability

The datasets used and/or analysed during the current study are available from the corresponding author on reasonable request.

## Abbreviations

OW: Office Worker
PA: Physical activity
HEPA: Health-enhancing physical activity
HRQOL: Health-related quality of life
PAHCO: PA-related health competence
WHPP: Workplace health promotion program
PASR: PA-specific self-regulation
CCPT: Control competence for physical training
WHO: World Health Organisation
PAAR: PA-specific affect regulation
IRB: Institutional review board
DRKS: Deutsches Register Klinischer Studien
GSLTPAQ: Godin-Shepard leisure-time physical activity questionnaire
LSI: Leisure score index
SF-12: Short-form health survey
PP: Per-protocol
MA: Massachusetts
USA: United States of America
LMM: Linear mixed model
AIC: Akaike’s information criteria
BIC: Bayesian information criteria
CI: Confidence interval
M: Mean
SD: Standard deviation
N: Total sample
n: Subsample
