# Supplementary material for: Promoting physical activity-related health competence to increase leisure-time physical activity and health-related quality of life in German private sector office workers
Source: BMC Public Health. 2023 Mar 11;23:470. doi: 10.1186/s12889-023-15391-7 (PMC10007852; doi:10.1186/s12889-023-15391-7)
Supplement: Supplementary file 1 — Additional file 1: Supplementary Table 1. Model-fit parameters for null model and the final regression models. Supplementary Fig. 1. Physical activity-related health competence over time. Supplementary Fig. 2. Control competence for physical training over time. Supplementary Fig. 3. Physical acitivity-specific affect regulation over time. Supplementary Fig. 4. Physical acitivity-specific self-regulation over time. [file 12889_2023_15391_MOESM1_ESM.pdf]

Supplementary Material

Supplementary Table 1. Model-fit parameters for null model and the final regression models

Criterion	Model	*DF*	*BIC*	*AIC*	*adj. R^2*
PAHCO	lmer(PAHCO ~ 1 + (1 | ID))	3	786.07	772.89	0.35
	lmer(PAHCO ~ Gender + Age + Relationship Status + Education Level + Leisure-time PA + Measurement time + (1 | ID))	12	595.53	542.81	0.65
CCPT	lmer(CCPT ~ 1 + (1 | ID))	3	942.50	929.33	0.26
	lmer(CCPT ~ Gender + Age + Relationship Status + Education Level + Leisure-time PA + Measurement time + (1 | ID))	12	766.58	713.92	0.60
PAAR	lmer(PAAR ~ 1 + (1 | ID))	3	899.21	846.55	0.54
	lmer(PAAR ~ Gender + Age + Relationship Status + Education Level + Leisure-time PA + Measurement time + (1 | ID))	12	903.58	890.41	0.63
PASR	lmer(PASR ~ 1 + (1 | ID))	3	1156.75	1169.91	0.38
	lmer(PASR ~ Gender + Age + Relationship Status + Education Level + Leisure-time PA + Measurement time + (1 | ID))	12	1022.58	1075.25	0.57
Leisure-time PA	lmer(Leisure-time PA ~ 1 + (1 | ID))	2	1700.31	1691.54	0.00
	lmer(Leisure-time PA ~ Gender + Age + Relationship Status + Education Level + Measurement time + HRQOL + CCPT + PAAR + PASR + (1 | ID))	15	1306.29	1240.47	0.65
HRQOL	lmer(HRQOL ~ 1 + (1 | ID))	2	1700.31	1691.54	0.00
	lmer(HRQOL ~ Gender + Age + Relationship Status + Education Level + Measurement time + Leisure-time PA + CCPT + PAAR + PASR + (1 | ID))	15	1413.62	1347.80	0.63

Notes. DF = Degrees of Freedom, BIC = Bayesian Information Criterion, AIC = Akaike Information Criterion, adj. R2 = Corrected Proportion of Explained Variance, PAHCO = Physical Activity-related Health Competence, CCPT = Control Competence for Physical Training, PAAR = Physical Activity-specific Affect Regulation, PASR = Physical Activity-specific Self-Regulation

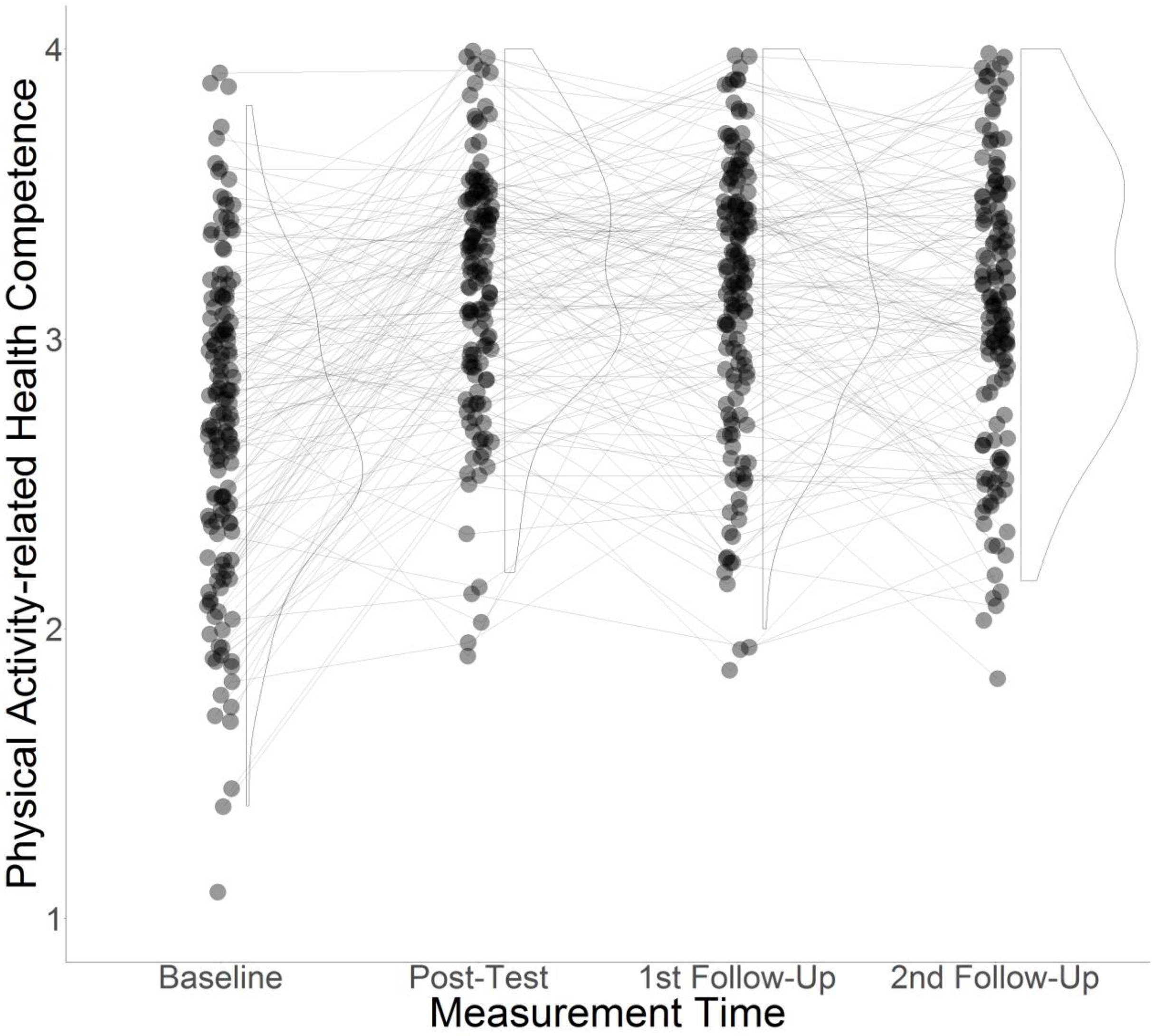

Supplementary Figure 1. Physical activity-related health competence over time

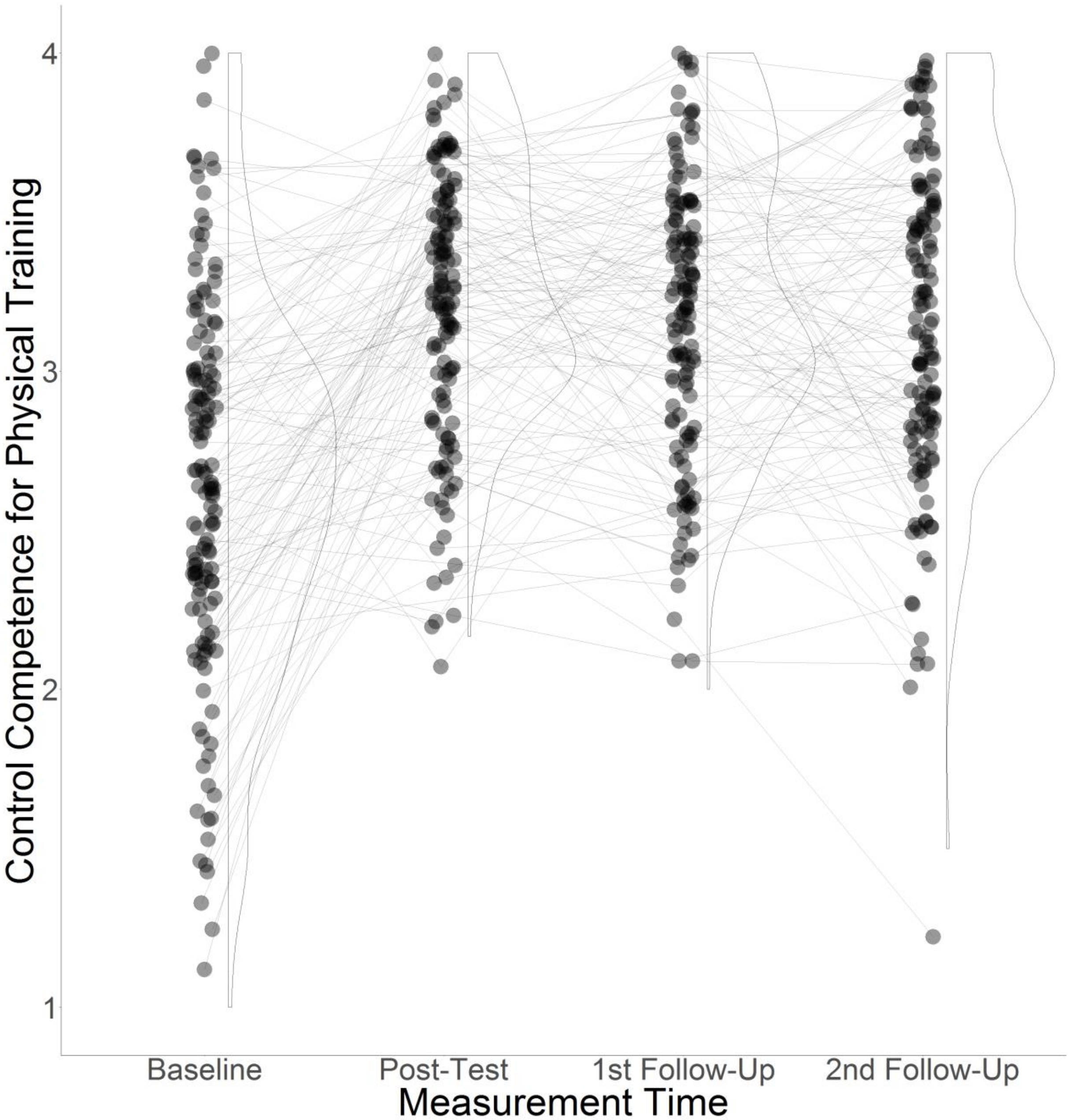

Supplementary Figure 2. Control competence for physical training over time

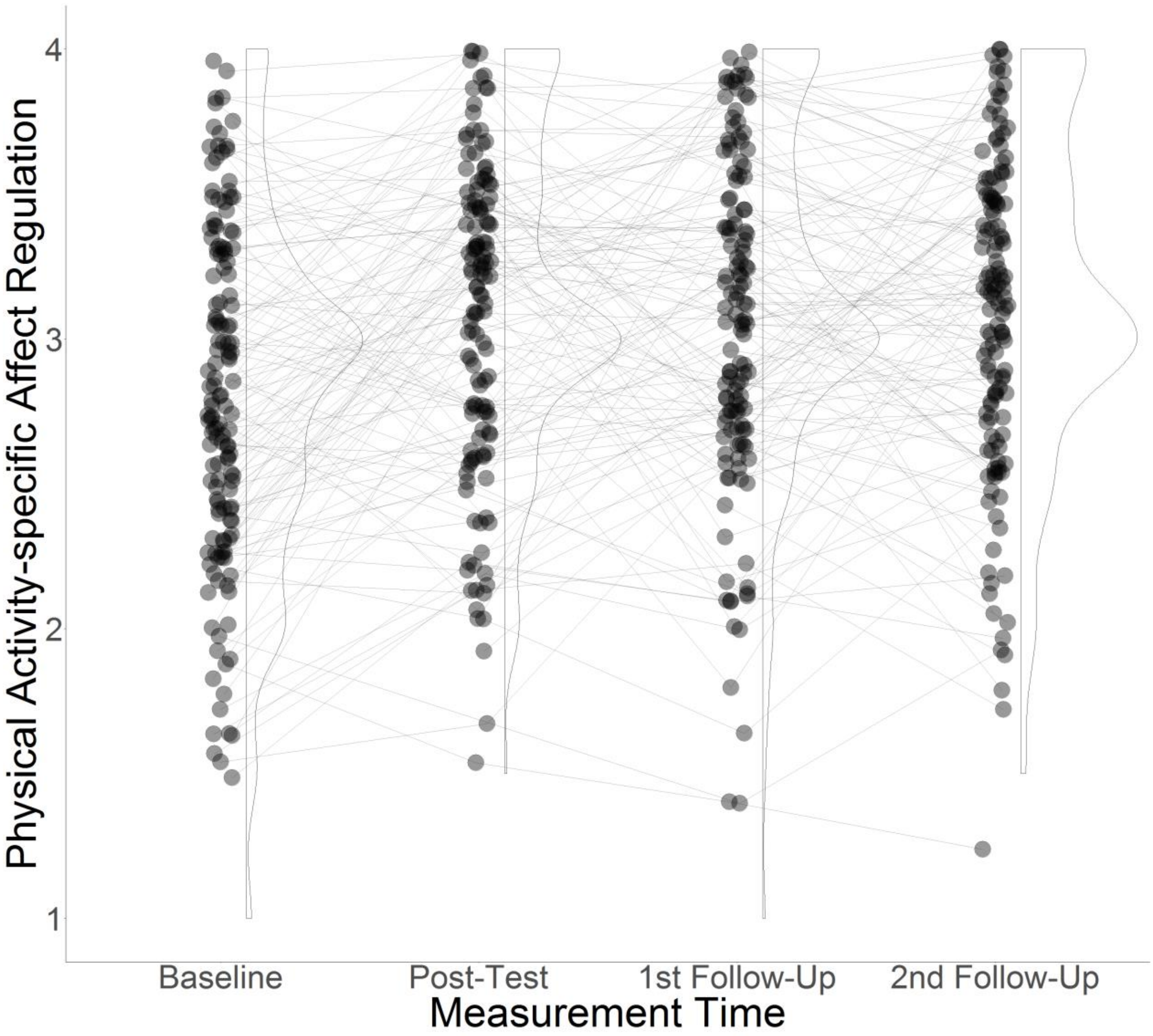

Supplementary Figure 3. Physical acitivity-specific affect regulation over time

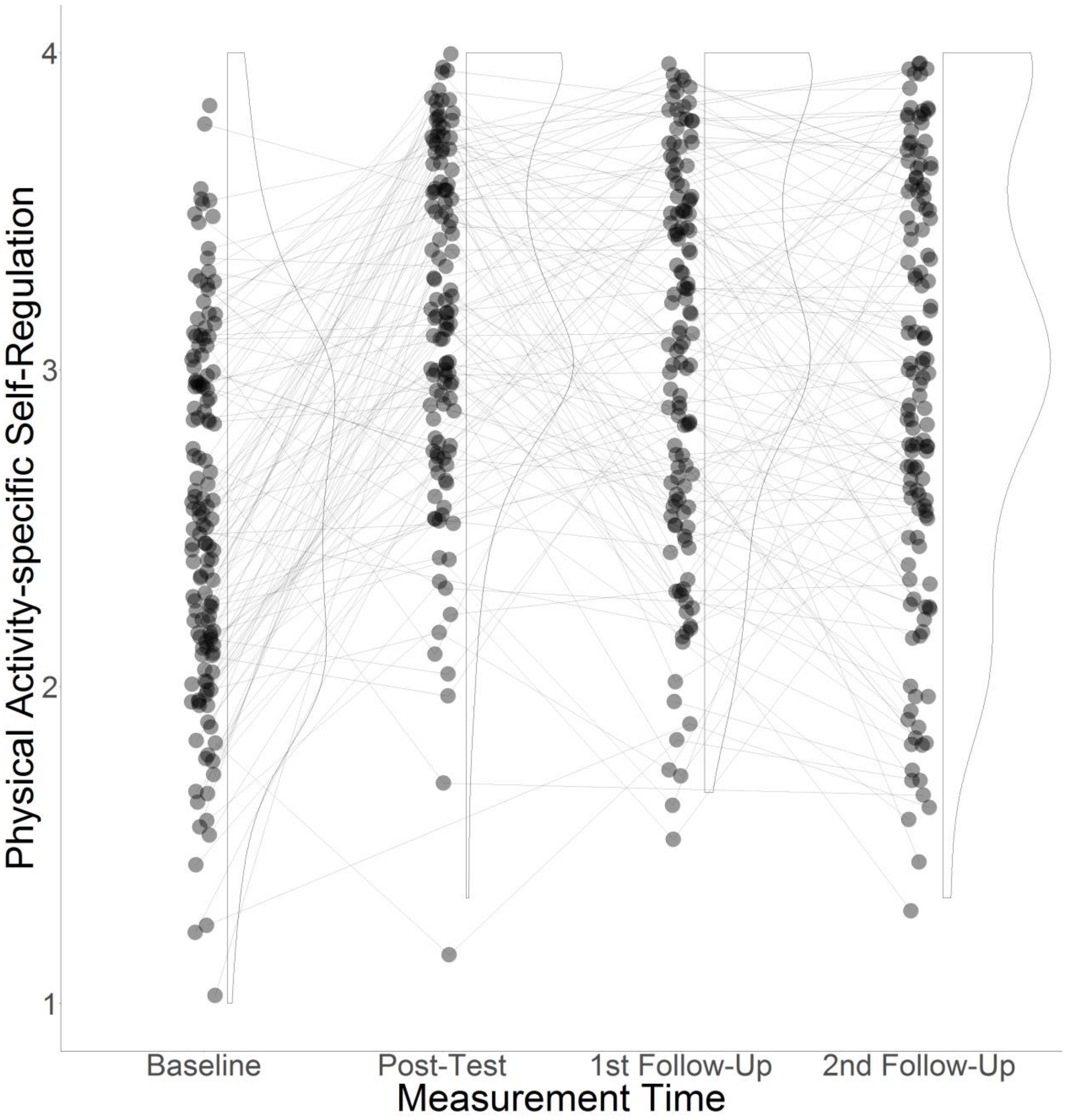

Supplementary Figure 4. Physical acitivity-specific self-regulation over time
